# Catching a SPY: Using the SpyCatcher-SpyTag and Related Systems for Labeling and Localizing Bacterial Proteins

**DOI:** 10.3390/ijms20092129

**Published:** 2019-04-30

**Authors:** Daniel Hatlem, Thomas Trunk, Dirk Linke, Jack C. Leo

**Affiliations:** Bacterial Cell Surface Group, Section for Evolution and Genetics, Department of Biosciences, University of Oslo, 0316 Oslo, Norway; daniel.hatlem@ibv.uio.no (D.H.); thomas.trunk@ibv.uio.no (T.T.); dirk.linke@ibv.uio.no (D.L.)

**Keywords:** autotransporter, covalent labeling, bacterial surface protein, SpyCatcher, topology mapping, virulence factor

## Abstract

The SpyCatcher-SpyTag system was developed seven years ago as a method for protein ligation. It is based on a modified domain from a *Streptococcus pyogenes* surface protein (SpyCatcher), which recognizes a cognate 13-amino-acid peptide (SpyTag). Upon recognition, the two form a covalent isopeptide bond between the side chains of a lysine in SpyCatcher and an aspartate in SpyTag. This technology has been used, among other applications, to create covalently stabilized multi-protein complexes, for modular vaccine production, and to label proteins (e.g., for microscopy). The SpyTag system is versatile as the tag is a short, unfolded peptide that can be genetically fused to exposed positions in target proteins; similarly, SpyCatcher can be fused to reporter proteins such as GFP, and to epitope or purification tags. Additionally, an orthogonal system called SnoopTag-SnoopCatcher has been developed from an *S. pneumoniae* pilin that can be combined with SpyCatcher-SpyTag to produce protein fusions with multiple components. Furthermore, tripartite applications have been produced from both systems allowing the fusion of two peptides by a separate, catalytically active protein unit, SpyLigase or SnoopLigase. Here, we review the current state of the SpyCatcher-SpyTag and related technologies, with a particular emphasis on their use in vaccine development and in determining outer membrane protein localization and topology of surface proteins in bacteria.

## 1. Introduction

**Figure 1:**
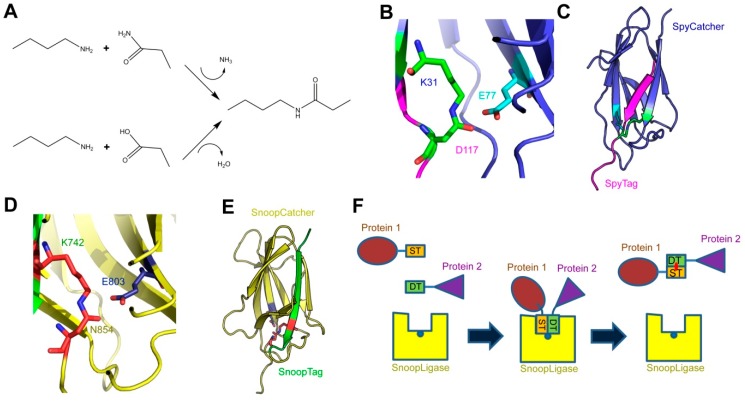
The SpyCatcher-SpyTag, SnoopCatcher-SnoopTag, and SnoopLigase systems. (**A**) Formation of an isopeptide bond. The primary amine of a lysine side chain condenses with either the side-chain amide of an asparagine (upper reaction) or the side-chain carboxyl of an aspartate (lower reaction) to produce the isopeptide bond, releasing either ammonia or water, respectively; (**B**) the isopeptide bond between SpyCatcher-SpyTag. The isopeptide formed by the reactive lysine (K31) in SpyCatcher and aspartate (D117) in SpyTag is shown in green, and the catalytic glutamate (E77) in cyan; (**C**) crystal structure of SpyCatcher-SpyTag. SpyCatcher is in blue and SpyTag is in magenta. The structures shown in panels b and c are based on the Protein Data Bank (PDB) entry 4MLI [20]; (**D**) the isopeptide bond between SnoopCatcher-SnoopTag, formed by lysine (K742) in SnoopTag and the asparagine (N854) in SnoopCatcher, is shown in red, and the catalytic glutamate (E803) is in blue; (**E**) crystal structure of SnoopCatcher-SnoopTag. SnoopCatcher is in yellow and SnoopTag in green. The structures shown in panels d and e are based on the RrgA D4 domain structure (PDB ID: 2WW8) [15]; (**F**) schematic of the function of SnoopLigase. A protein (in brown) containing SnoopTagJr (ST, in orange) and another protein (purple) with DogTag (DT, green) are mixed in the presence of SnoopLigase (yellow). The tags bind to SnoopLigase, which contains the catalytic glutamate (blue dot) and catalyzes the formation of an isopeptide between SnoopTagJr and DogTag (red dot). The ligated proteins can then be eluted from SnoopLigase.

The peptide bond is essential to biology. This secondary amide bond is produced by the condensation of the carboxyl group of one amino acid residue with the α-amino group of the following residue and results in the polymerization of amino acids to produce polypeptides. This central reaction is catalyzed by the ribosome and the sequence of the polypeptide chain is defined by the genetic code. In proteins, the peptide bond is sometimes referred to as the eupeptide bond. In addition, peptide bonds can be found in non-ribosomal peptides, which are secondary metabolites such as antibiotics produced by non-ribosomal peptide synthases [1]. A minority of proteins contain a second class of peptide bonds, referred to as isopeptide bonds. This is an amide bond similar to the peptide bond, but it is produced by the condensation of an amine group with a carboxyl group or a primary amide group located in amino acid side chains, rather than the main chain-building amino or carboxyl groups (Figure 1A).

In eukaryotes, the most prevalent example of isopeptide bond formation is the ubiquitination of proteins targeted for degradation by the 26S proteasome [2]. The isopeptide is formed by the C-terminal carboxyl group of ubiquitin and the side-chain amine of a lysine residue in the target protein. This reaction is catalyzed by E3 enzymes, or ubiquitin-protein ligases, which also impart specificity to the system [3]. Similarly, members of the small ubiquitin-like modifiers (SUMO) family, which regulate a number of cellular processes, are ligated to their target proteins via an isopeptide bond [4].

In bacteria, isopeptides are mostly known from surface proteins of Gram-positive bacteria. Two classes of proteins have notable isopeptide bonds: the microbial surface components recognizing adhesive matrix molecules (MSCRAMMs) and pili [5]. In pili, pilin subunits are covalently bound to each other via an intermolecular isopeptide bond between the carboxyl group of a C-terminal threonine and the ε-amine of a lysine in the next subunit, a reaction catalyzed by a pilin-specific sortase [6]. In MSCRAMMs and some pilins, isopeptides are formed spontaneously between residues within the same domain. These domains belong to the immunoglobulin fold superfamily. Isopeptide bond formation increases the stability of the proteins or pili and may contribute to stronger binding to ligands by MSCRAMMs, since in some cases the isopeptide only forms upon ligand binding to lock the protein in a bound conformation [7].

The SpyCatcher-SpyTag system was developed by the Howarth laboratory based on the internal isopeptide bond of the CnaB2 domain of FbaB, a fibronectin-binding MSCRAMM and virulence factor of *Streptococcus pyogenes* [8,9]. An internal isopeptide bond forms spontaneously in this domain between the ε-amine of lysine K31 and the side chain carboxyl of aspartic acid D117. The reaction is catalyzed by the spatially adjacent glutamate E77 (Figure 1B). The resulting isopeptide bond confers high stability to the CnaB2 domain [10]. The CnaB2 domain can be stably split into two components: a larger, incomplete immunoglobulin-like domain (termed SpyCatcher) of 138 residues (15 kDa) and a shorter peptide (SpyTag) of 13 residues (see Table 1 for peptide sequences). SpyCatcher contains the reactive lysine and catalytic glutamate, whereas SpyTag includes the reactive aspartate. The two components can still recognize each other with high affinity (0.2 µM) and the isopeptide can form between SpyCatcher and SpyTag to form a covalently bound complex (Figure 1C). Under experimental conditions relevant to life science research (room temperature, dilute protein concentrations), the reaction rates allow the bonds to form at high efficiency within minutes [9]. SpyTag in particular is equivalent in size to a number of epitope tags and can be genetically fused to a number of proteins and is able to react with SpyCatcher when inserted at the N- or C-termini of target proteins, as well as internal sites [9,11]. SpyCatcher itself can also be produced as a fusion protein, allowing the formation of covalently bonded protein partners that might otherwise be difficult to produce as protein fusions [12,13,14].

A similar system has been developed based on another Gram-positive surface protein, the pilus adhesin RrgA of *S. pneumoniae* [15,16]. The D4 domain of this protein is stabilized by an isopeptide forming between a lysine (K742) and an asparagine (N854), catalyzed by the spatially adjacent E803 [15] (Figure 1D). This domain was split into a scaffold protein called SnoopCatcher and a 12-residue peptide termed SnoopTag, which can spontaneously form a covalent isopeptide bond upon mixing [16] (Figure 1E). In contrast to SpyCatcher-SpyTag, the reactive lysine is present in SnoopTag and the asparagine in SnoopCatcher. This system is orthogonal to SpyCatcher-SpyTag; that is, SnoopCatcher does not react with SpyTag and SpyCatcher does not react with SnoopTag. This allows the use of both systems simultaneously to produce “polyproteams,” programmed modular polyproteins, for use in biotechnological applications [16].

The Howarth lab further modified these technologies by making tripartite systems, where the isopeptide-forming lysine and aspartate/asparagine are located on separate peptides, and the catalytic glutamate is present on a larger scaffold protein [17]. This was first attempted with the SpyCatcher system to produce SpyLigase. Here, a second peptide containing the reactive lysine (KTag) was separated from SpyCatcher, which itself was modified to produce the stable SpyLigase protein containing the catalytic glutamate [18]. When SpyLigase was mixed with two proteins containing one of the reactive peptides each, SpyLigase was able to catalyze the fusion of the two tags. However, although SpyLigase could mediate the fusion of KTag and SpyTag located at both N- and C-terminal and even internal positions, the reactions had a ~50% efficiency at best and were dependent on specific buffer conditions and low temperature [16]. In contrast, the recently developed SnoopLigase system appears more robust and efficient [19]. SnoopLigase was engineered similarly to SpyLigase, and catalyzes the isopeptide formation between the lysine of a modified SnoopTag (SnoopTagJr) and the asparagine in a second peptide termed DogTag (Figure 1F). This system can have efficiencies over 95% that are less sensitive to temperature and reaction conditions than SpyLigase. Furthermore, as SnoopTagJr and DogTag have relatively high affinity for SnoopLigase, immobilizing SnoopLigase allows washing away unconjugated reactants followed by elution of essentially pure fusion products [19]. The various Catcher-Tag systems and their development are summarized in Table 1.

Below, we review the various applications of the SpyCatcher/SnoopCatcher systems in biotechnology. Our emphasis will be on using this methodology to label surface-exposed proteins, especially for mapping the topology of outer membrane-embedded virulence factors of Gram-negative bacteria.

## 2. Applications of the SpyCatcher-SpyTag System

Being a quick and reliable coupling tool for irreversible peptide-protein ligation, the SpyTag-SpyCatcher system is ideal for a wide range of applications, ranging from increasing protein stability to antigen delivery during vaccination [14,23]. Before focusing on the use of the SpyTag-SpyCatcher system in the investigation of bacterial virulence factors, we give a short overview of those applications. For more detailed information, a list of publications and patents using the SpyTag-SpyCatcher and related technologies is accessible at the SpyInfo web page (available online: https://www.bioch.ox.ac.uk/howarth/info.htm) and the corresponding sequences and expression routes are listed in the SpyBank database [24].

**Figure 2:**
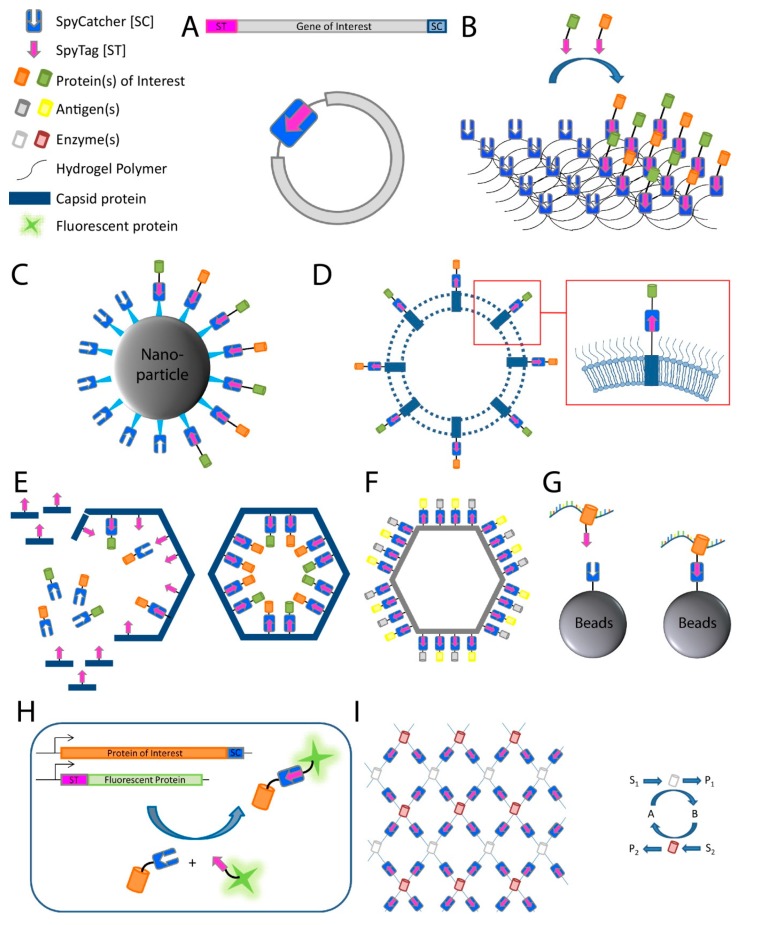
Applications of the SpyCatcher-SpyTag system. (**A**) SpyRing: SpyCatcher and SpyTag are fused to the terminal ends of the protein of interest resulting in protein cyclization and thereby conferring an increased resilience to denaturation; (**B**) post-hydrogelation decoration of protein hydrogels: SpyCatcher is fused to the polymeric material used in hydrogel synthesis, which allows the posthydrogelation decoration with proteins of interest fused to SpyTag; (**C**–**F**): (**C**) bioconjugation of target proteins to nanoparticles, (**D**) outer membrane vesicles, (**E**) phage capsid proteins to create a proteinaceous nanocompartment, and (**F**) virus-like particles; (**G**) SpyCLIP: SpyTagged RNA-binding protein interacts with RNA and is covalently attached to beads with fused SpyCatcher for use in pull-down assays; (**H**) fluorescent protein labeling for use, for example, in microscopy (ST represents SpyTag sequence); (**I**) artificial multi-enzyme nanodevices for increased efficiency, stability, and reusability. Schematic of mesh-like nanodevice is shown on the left and an enzymatic reaction scheme on the right. S represents substrate; P represents product; A and B represent cofactors.

A major application of the SpyTag-SpyCatcher system is the formation of so-called SpyRings in order to increase the intrinsic resilience of proteins to denaturation. SpyRings are generated by circularization of a single protein, which is accomplished by fusing an N-terminal SpyTag with a C-terminal SpyCatcher, or vice versa (Figure 2A). Enzyme circularization increases resistance to hyperthermal denaturation and aggregation, as well as alkali tolerance of individual enzymes without a loss in enzymatic activity [25,26,27,28,29]. Enzymes with a short distance between termini (<15 Å), and an active site which is not in direct proximity to one of the termini, are considered ideal candidates for protein circularization [30]. However, efficient SpyRing circularization and increased thermal stability have been shown for proteins where the termini are even farther apart [28].

Another frequently used application for the SpyCatcher-SpyTag system is the decoration of protein hydrogels (Figure 2B). Hydrogels have found application, for example as an artificial extracellular matrix material in medicine [31], tissue engineering [32,33], and cell culturing [34]. Hydrogels are polymeric materials engineered to resemble the extracellular environment of specific tissues with defined functional and structural properties. They have been used for decades as molecule delivery devices and as carriers for cells in tissue engineering due to their ability to mimic aspects of the native cellular environment [35]. However, hydrogels fail to fully imitate the complexity of biological systems. Fusing SpyCatcher to the polymeric material used in hydrogel synthesis allows the decoration with SpyTagged proteins post-hydrogelation to mimic specific microenvironments [36,37,38,39]. Thus, the SpyTag-SpyCatcher system is used as a quick and simple molecular tool for the simultaneous incorporation and presentation of different target molecules into and on hydrogels, thereby avoiding an otherwise laborious engineering process.

The SpyTag-SpyCatcher system has also been used for the modular assembly of proteins onto nanoparticles [40] (Figure 2C) and bacterial outer membrane vesicles [41] (Figure 2D; see also Section 3.3). The same system can be used for the in vivo encapsulation of enzymes fused to phage capsid proteins in order to create a protein nanocompartment [42] (Figure 2E) and for the decoration of virus-like particles (VLPs) for antigen delivery to the immune system [43,44] (Figure 2F; see also Section 3.3), while successfully preserving the structure and the function of the assembled proteins.

Recently, the SpyTag-SpyCatcher system was used for the characterization of protein–RNA interactions as an alternative for ultraviolet (UV) crosslinking and immunoprecipitation (CLIP) [45]. The CLIP method relies on the limited specificity of antibody–antigen interactions which cannot withstand harsh washing conditions resulting in insufficient purity of ribonucleoprotein complexes after immunoprecipitation. Therefore, additional gel purification steps are necessary to further purify these complexes, which results in loss of protein. The disadvantages of CLIP are the labor-intensive gel purification steps as well as a high number of false-positive signals due to non-specific interactions. In comparison, the SpyTag-SpyCatcher technology allows the method to be performed with beads, skips the gel purification steps altogether, and withstands harsh washing steps, thus reducing non-specific interactions. The improved method using the Spy technology was termed SpyCLIP and requires SpyCatcher being fused to beads for immunoprecipitation and fusion proteins of the protein of interest, in this case the RNA-binding protein, to SpyTag [45] (Figure 2G). UV-crosslinking and affinity purification (uvCLAP) [46] as well as gel-omitted ligation-dependent CLIP (GoldCLIP) [47], using the covalent HaloTag-HaloLink purification system for immunoprecipitation [48], are both improvements to the traditional CLIP protocol, omitting the labor-intensive gel purification steps. The advantage of the SpyCatcher system over those methods is the ability of SpyTag-SpyCatcher to withstand even harsher washing conditions than the biotin-streptavidin coupling used during uvCLAP, and the small size of SpyTag compared to the 33 kDa protein tag used in GoldCLIP.

The system has also found increasing usage in conventional and super-resolution microscopy [49,50,51] by providing an easy and reliable way to label a SpyTagged protein with SpyCatcher coupled to a fluorescent dye (Figure 2H). Additionally, the limited accessibility of large antibodies, classically used for detection of epitope-tagged molecules, can be avoided by using the relatively small SpyCatcher protein during the detection of SpyTagged targets.

The Spy technology can also be used for the targeting of chemically synthesized voltage-sensitive dyes to specific cells for optical measurement of voltage dynamics in living cells. To this end, SpyTag is linked via a polyethylene linker to voltage-sensitive dyes while expressing SpyCatcher on the target cells. These SpyTag–dye conjugates, termed Voltage Spy, display improved targeting, good voltage sensitivity, and fast-response kinetics [52].

The system can also be used for the spatial and functional coordination of enzyme functions, creating a microenvironment normally associated with biological compartmentalization and conferring some of the same benefits (e.g., a local increase in enzyme and metabolite concentrations). Linkage of SpyTag with SpyCatcher is utilized for enzyme organization, allowing construction of artificial multi-enzyme nanodevices with a controlled spatial arrangement for increased efficiency of enzyme cascades while maintaining or even increasing stability and reusability of biocatalysts [53] (Figure 2I).

## 3. Using SpyCatcher-SpyTag to Investigate Bacterial Virulence Factors

The SpyCatcher-SpyTag system has recently been used for investigating bacterial virulence factors. So far, these have been limited to studying surface proteins of Gram-negative bacteria, especially autotransporter proteins. Autotransporters, also called type V secretion systems, are a widespread family of secreted proteins from Gram-negative bacteria, and many of these mediate virulence-related functions [54]. These proteins, which are divided into several subclasses (type Va through type Ve), have an outer membrane-embedded β-barrel domain and an extracellular region or passenger, which harbors the specific activity of each protein. Although SpyCatcher-SpyTag has been used mostly for studying autotransporters, the methods described below would be applicable to other surface-exposed proteins, both in Gram-negative and Gram-positive bacteria.

The cell surface of Gram-negative bacteria is represented by the outer membrane (OM), which is a complex asymmetric lipid bilayer. The extracellular face of the OM is mainly composed of lipopolysaccharides and outer membrane proteins that include several common virulence factors responsible for adhesion, mobility, and secretion, such as autotransporter adhesins, flagella, and type I-VIII secretion systems, among others [55,56]. Studying the expression, secretion, migration, and interactions of OM proteins often requires labeling by reporter proteins or other fluorophores. Labeling OM proteins, however, is challenging. Many fluorescent reporter proteins fail to mature in the periplasm [57]; thus, genetic fusions with fluorescent proteins are limited to a handful of options [58,59,60,61]. An alternative approach is non-covalent affinity-based labeling by using antibody–reporter protein fusions, affinity tags, or other high-affinity interactions, for example, colicins ColE9 and ColIa for labeling of the vitamin B12 transporter BtuB [62]. Alternatively, labeling methods using small organic molecules include amine-reactive fluorescence labeling [63,64], cysteine-reactive labeling [65], or site-specific labeling with unnatural amino acids, and tag-specific labeling [66,67]. Each of these techniques, however, has shortcomings. Non-covalent labeling requires high (nM) affinities for the label to remain associated with its target for a significant amount of time, which often makes such labeling unsuitable for time-resolved imaging on the minute scale. Small-molecule labeling often requires harsh treatment of the cells with reactive molecules, and site-specific orthogonal labeling requires co-expression of additional OM proteins that can disturb the system. In this section, we present a few recent examples showing how the SpyCatcher system can overcome many of these challenges by covalently labeling OM-bound virulence factors with high specificity, both with the purpose of studying the virulence factors and of exploiting their properties in vaccine development.

### 3.1. Using SpyCatcher to Investigate Membrane Protein Topology and Secretion

**Figure 3:**
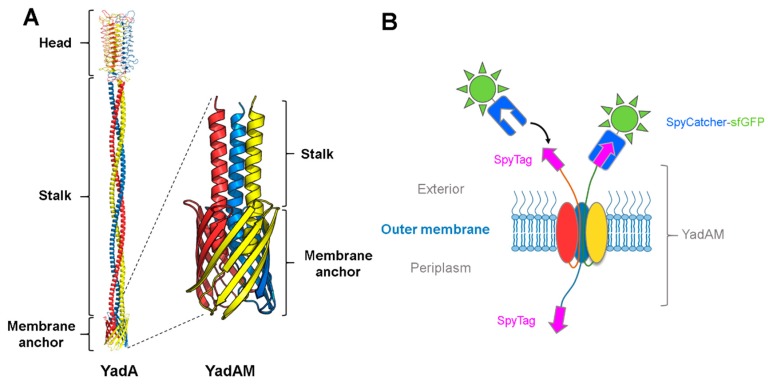
Using SpyCatcher to determine the surface topology of the *Yersinia* adhesin YadA. (**A**) Model of full-length YadA [71] and solid-state NMR structure of YadAM (PDB ID: 2LME); (**B**) schematic of the trimeric SpyTag-YadAM_A354P_ construct with two secreted chains. The secreted chains fused to SpyTag are located on the bacterial surface and are able to bind to the SpyCatcher-sfGFP, while the chain located in the periplasm is inaccessible to the SpyCatcher fusion.

Trimeric autotransporter adhesins (TAAs), or type Vc secretion systems, constitute a group of surface-displayed virulence factors in Gram-negative bacteria that are responsible for adhesion to organic and inorganic surfaces. The prototypical TAA is *Yersinia* adhesin A (YadA), which consists of a C-terminal transmembrane β-barrel domain, where three protomers form a 12-stranded β-barrel, and an N-terminal trimeric passenger that is translocated through the β-barrel to the bacterial surface [68]. Once the passenger is translocated, it adopts a lollipop-like structure, forming a fibrous trimeric coiled-coil stalk that ends in an N-terminal globular head domain, responsible for adhesion and autoaggregation (Figure 3A). Autotransporters belonging to the type Va and Ve secretion systems initiate their translocation process via a hairpin intermediate, meaning that the part of the passenger most proximal to the β-barrel is initially inserted into the pore and then exported in a hairpin-like loop from the β-barrel until the entire passenger is outside the cell [69,70].

**Figure 4:**
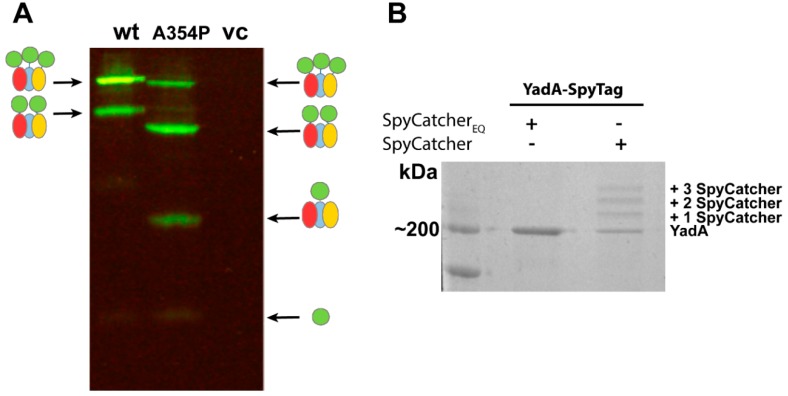
SpyCatcher assays with YadA. (**A**) Semi-native SDS-PAGE of OM preparations of wild-type SpyTag-YadAM and SpyTag-YadAM_A354P_ after treatment with SpyCatcher-sfGFP, visualized under blue light. The ladder-like pattern indicates 1–3 bound SpyCatcher-sfGFP molecules. The gel shown is based on results from [13]. The wild-type YadAM binds mainly three SpyCatcher-sfGFP molecules, demonstrating full surface exposure of the N-terminal SpyTags, whereas the A354P mutant mainly binds to 1–2 SpyCatcher-sfGFP molecules. Note that YadAM_A354P_ migrates anomalously compared with the wild-type. A schematic of the species visible in the gel is given on the side: SpyCatcher-sfGFP in green and YadAM monomers in red, blue, and yellow. vc represents vector control; (**B**) SDS-PAGE of OM preparations of full-length, trimeric SpyTag-YadA (~200 kDa) treated with the inactive SpyCatcher_EQ_ and normal SpyCatcher, stained with Coomassie blue. The SpyCatcher-treated SpyTag-YadA shows a similar ladder-like pattern as in panel a, corresponding to the change in electrophoretic mobility as 1–3 SpyCatchers are bound.

In a recent study, we investigated whether the hairpin model also holds true for TAAs using YadA as a model system, and aided by the SpyCatcher-SpyTag technology [13]. Earlier studies had identified a flexible region at the start of the coiled coil embedded within the lumen of the β-barrel, termed the ASSA region, due to the sequence consisting of alanines and serines. The ASSA region was hypothesized to be important for the formation of the hairpin [72]. To test this hypothesis, a truncated YadA construct was used, containing only the β-barrel and a small part of the passenger, termed YadAM (for YadA membrane anchor; see Figure 3A). Single proline substitutions were introduced to the ASSA region to probe its importance for translocation. Prolines are known secondary structure disruptors due to their rigid backbone, and we expected the substitutions to disrupt the flexible ASSA region sufficiently to stall the autotransporter process and trap the N-termini in the periplasm. To test this, a representative mutant, YadAM_A354P_, was chosen for further studies, and a SpyTag was inserted in the wild-type YadAM and YadAM_A354P_ constructs at the N-terminus of the mature proteins, following the signal peptide required for secretion. For easy detection, we fused purified SpyCatcher fused to sfGFP (superfolder green fluorescent protein). *Escherichia coli* cells expressing the different constructs were incubated with SpyCatcher-sfGFP (that is too large to penetrate the OM), allowing the SpyCatcher domain to bind to the exported SpyTags presented on the bacterial surface, but not to any stalled intermediates where the SpyTags would still be located in the periplasm. Bacterial cells producing the wild-type construct displayed higher fluorescence compared with cells producing the A354P mutant, indicating that the mutation was interfering with secretion of the passenger domain [13]. The effect of the A354P mutation on the surface topology of YadA was further investigated by isolating OM fractions of the two constructs after SpyCatcher-sfGFP treatment and analyzing them by semi-native SDS-PAGE (i.e., without heating the samples beforehand) (Figure 4A). GFP is stable in SDS at ambient temperatures [73], allowing instant visualization of GFP-containing bands by in-gel fluorescence under blue light. The gel showed a ladder-like pattern of SpyTag-YadAM complexes bound to 1–3 SpyCatcher-GFP molecules, corresponding to the number of strands exported to the bacterial surface (Figure 3B and Figure 4A). Comparison of the band intensities indicates that the majority of YadAM exports all three passengers, whereas YadAM_A354P_ mainly exports 1–2 SpyTags. These experiments demonstrated that the proline substitution is a partially stalled intermediate where one or two passenger strands are exported, but not a fully blocked autotransport intermediate.

As an alternative to using reporter molecules such as GFP to study the surface topology of OM proteins, the binding of SpyCatcher alone can be utilized for assessing surface exposure. Because SpyCatcher forms a covalent bond with SpyTag, the added molecular weight of SpyCatcher causes a change in electrophoretic mobility, giving a shift of 16 kDa that can be observed either by Coomassie staining or Western blotting. As an example, we studied the surface exposure of the full-length, SpyTagged YadA construct. YadA was treated with SpyCatcher alone (i.e., with no fusion partner) and compared with YadA treated with an inactive SpyCatcher, where the catalytic glutamate was changed to glutamine (SpyCatcher_EQ_), by SDS-PAGE. In this example, unlabeled YadA remains trimeric even when heated to 95 °C and is seen as a single band at 200 kDa [74]. By contrast, the sample treated with active SpyCatcher forms a ladder-like pattern corresponding to YadA alone and YadA plus 1–3 bound SpyCatchers (Figure 4B). The experiments with truncated and full-length YadA demonstrate how SpyCatcher can readily bind to SpyTags located in close proximity to the bacterial surface, as shown for the YadAM constructs, as well as for the more exposed SpyTag protruding far from the surface located on the full-length protein. Future work will determine whether this technology is also suitable for detecting OM proteins without protruding domains, for example, where SpyTag is inserted into the loops of transmembrane β-barrel domains.

### 3.2. Using SpyCatcher to Investigate Membrane Dynamics

The OM of Gram-negative bacteria consists of a mixture of OM proteins, lipopolysaccharides, and phospholipids. Both OM proteins and lipopolysaccharides are inserted into the OM in localized patches around the cell center where they diffuse to form a uniform distribution across the bacterial surface [75]. When the bacterium grows in preparation for cell division, the outer membrane proteins migrate towards the poles as a result of new membrane material and peptidoglycan being incorporated near the cell center in a process that is still not well understood [62].

**Figure 5:**
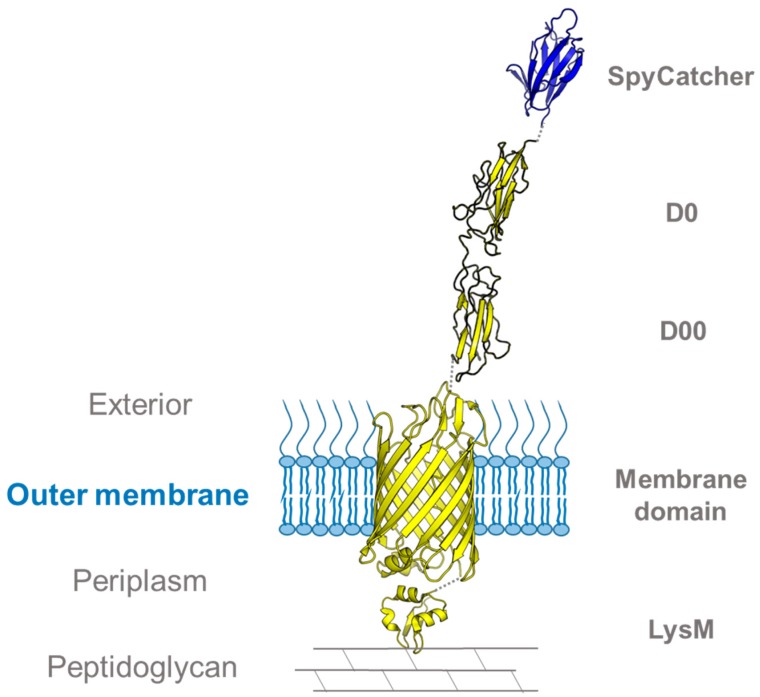
Intimin–SpyCatcher002 fusion for studying membrane dynamics. Structural model of the intimin construct used in [21]. The model is based on the crystal structures of the intimin transmembrane domain (PDB ID: 4E1S) and SpyCatcher (PDB ID: 4MLI), the solution structure of the peptidoglycan-binding LysM domain (PDB ID: 2MPW), and the homology model structures of the extracellular D00 and D0 domains [81].

In a recent paper, Keeble et al. used the SpyCatcher system to study the OM dynamics in *E. coli*. To this end, they used a novel phage display method to develop an improved SpyCatcher-SpyTag pair, termed SpyTag002 and SpyCatcher002 [21]. The rate constant of the improved 002 pair was increased by an order of magnitude compared to the original SpyCatcher–SpyTag interaction (2.0 × 10^4^ M^−1^ s^−1^ vs. 1.7 × 10^3^ M^−1^ s^−1^). The efficiency of the newly developed pair was demonstrated by labeling the peptidoglycan-binding autotransporter intimin in order to study the membrane dynamics during cell growth and division. Intimin, a virulence factor of enterohemorrhagic *E. coli*, is a surface-exposed protein belonging to the type Ve secretion systems, also called inverse autotransporters [76]. Intimin consists of three major parts: an N-terminal periplasmic domain that binds to peptidoglycan under acidic conditions [77], a transmembrane β-barrel anchor [78], and the C-terminal passenger that is translocated to the bacterial surface through the β-barrel [79]. Keeble et al. prepared an intimin–SpyCatcher002 construct with SpyCatcher002 fused to the C-terminus of a truncated intimin variant (Figure 5). Upon overexpression in *E. coli* cells, the SpyCatcher002 domain is secreted together with the truncated passenger and presented on the bacterial surface, where it can be labeled using SpyTag002 fused to the fluorescent reporter protein mClover3 [80], in a similar fashion to that described for YadAM in Section 3.1. By imaging the cells using wide-field fluorescence microscopy after labeling, the movement of the mClover-tagged intimins toward the poles could be tracked to study the dynamics of the OM. Intimin was initially uniformly spread across the bacterial surface, but migrated towards the poles during cell division [21], suggesting that the polar movement results from incorporation of new peptidoglycan and OM material in preparation for cell division. This notion was further tested by repeating the experiment in the presence of cefalexin, a β-lactam antibiotic that prevents bacterial division by inhibiting septum formation and peptidoglycan synthesis. This time, the cells became elongated in preparation for division but were unable to divide. After 45 min, the fluorescent signal was localized in patches around the bacterium, thus demonstrating how inhibited peptidoglycan synthesis prevents the polar migration of OM proteins. Keeble et al. demonstrated here how an intimin–SpyCatcher fusion can be used to study the effect of peptidoglycan synthesis during division upon outer membrane dynamics [21]. This method could be used for tracking the migration of other OM proteins in the membrane during different stages of the cell cycle.

### 3.3. Exploiting Virulence Factors and Virus-Like Particles for Vaccine Development using SpyCatcher-SpyTag

Outer membrane vesicles (OMVs) are ubiquitously produced by Gram-negative bacteria and are often responsible for delivering virulence factors to the host cells during infection [82]. Derived from the outer membrane, OMVs contain many naturally occurring immunogenic components such as lipopolysaccharides, lipoproteins, outer membrane proteins, peptidoglycan, and other periplasmic components with intrinsic adjuvant properties [83]. As opposed to live and attenuated bacteria, OMVs are non-replicating and therefore pose no risk of infection after vaccination of immunocompromised individuals. Consequently, OMVs are promising candidates for modern vaccine development, both as adjuvants and as delivery vehicles for antigens. Vaccines utilizing OMVs as adjuvants have already been on the market for more than two decades; however, the development of heterologous antigen-presenting OMV-based vaccines using recombinant technology is still in its infancy [83,84]. Several strategies have been employed to present antigens on OMV surfaces, most notably as heterologous fusions with autotransporters [85,86,87]. The use of autotransporters for heterologous antigen presentation on OMVs has recently been reviewed in detail elsewhere [88], so we will only provide a short overview here.

**Figure 6:**
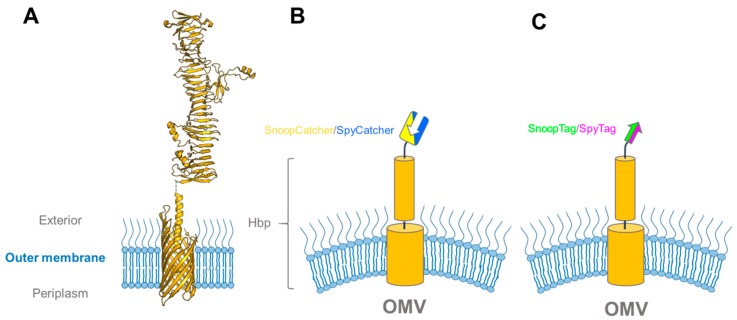
Hbp constructs used for vaccine display on outer membrane vesicles. (**A**) Structural model of full-length HbpD(Δd1) based on the crystal structures of the Hbp transmembrane domain (PDB ID: 3AEH) and passenger (PDB ID: 1WXR); (**B**) and (**C**) schematic drawings of Hbp fusions used in [92] to determine binding efficiencies between the SpyTag-SpyCatcher and SnoopTag-SnoopCatcher pairs on Hbp-fusion-presenting OMVs.

The Luirink group has pioneered the use of recombinant OMVs for vaccine development. As a scaffold, they earlier utilized a classical (type Va) autotransporter, the *E. coli* hemoglobin protease (Hbp). Hbp consists of two major parts: a C-terminal transmembrane β-barrel that is inserted into the outer membrane, and an N-terminal passenger that is translocated through the β-barrel to the bacterial surface where it is released to the environment through autoproteolysis (Figure 6) [89,90]. The Luirink group developed an Hbp variant suitable for heterologous antigen export and display by introducing a point mutation that prevents autoproteolysis into a truncated Hbp passenger domain (HbpD(Δd1); from now referred to only as Hbp) [86]. By combining the new Hbp variant with a hypervesiculating *Salmonella* Typhimurium Δ*tolRA* strain, they developed a method to isolate large amounts of Hbp-presenting OMVs [87]. To utilize Hbp as an antigen-presenting tool, several loops in the passenger were identified that allowed the insertion of heterologous protein antigens without impeding the autotransport process. However, even though the autotransporter accepts substantial changes to its passenger, there are still considerable limitations to the size and folding properties of passenger fusions [91].

**Figure 7:**
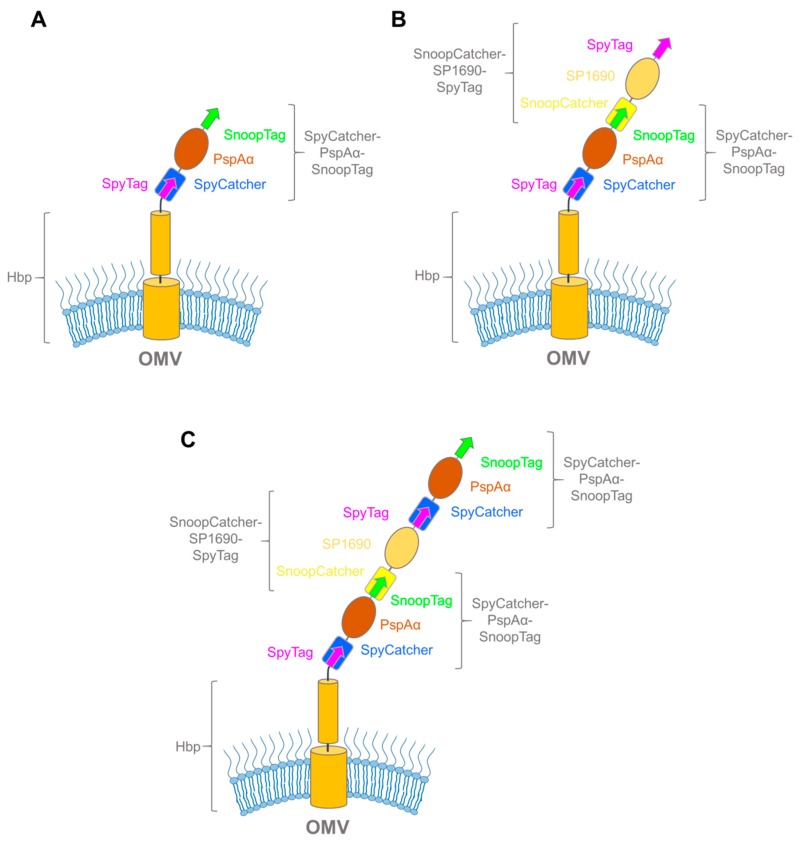
Hbp-antigen complexes made through Spy/SnoopCatcher fusions. (**A**) Schematic of Hbp bound to SpyCatcher–PspAα–SnoopTag; (**B**) schematic of Hbp bipartite binding adduct SpyCatcher–PspAα–SnoopTag and SnoopCatcher–SP1690–SpyTag; (**C**) schematic of Hbp tripartite binding adduct with two SpyCatcher–PspAα–SnoopTags flanking SnoopCatcher–SP1690–SpyTag.

In a recent paper, this problem was circumvented by using the SpyCatcher-SpyTag technology to decorate the OMVs after expression of Hbp [92]. In the process, the investigators comprehensively tested the applicability of the SpyCatcher and SnoopCatcher systems for modular vaccine building, and we will only cover the highlights here. To fully test the range of the new technology, the authors prepared OMVs presenting Hbp N-terminal fusions with SpyTag, SpyCatcher, SnoopTag, and SnoopCatcher (Figure 6). By treating the different constructs with their respective binding partners, and comparing the changes in electrophoretic mobility upon ligation by SDS-PAGE, they showed that all four Hbp-variants exported their passenger to the surface and were amenable to ligation. As a proof-of-concept for vaccine development, they proceeded to treat Hbp-SpyTag-presenting OMVs with a relatively large (24 kDa) domain of the antigenic *S. pneumoniae* surface protein, PspAα, fused to both SpyCatcher and SnoopTag (SpyCatcher–PspAα–SnoopTag) (Figure 7A). Earlier attempts to fuse PspAα with the Hbp passenger required the antigen to be divided into two smaller domains, which still resulted in significantly reduced export and lower antigen presentation on the OMV surface [93]. The SpyCatcher–PspAα–-SnoopTag, on the other hand, was ligated to the Hbp-presenting OMVs with similar efficiency as the earlier controls, demonstrating that the SpyCatcher system is a robust tool suitable for coating OMVs with recombinant antigens, completely circumventing the passenger export problem.

The next step was to check whether multiple antigens could be coupled to the OMV surface in an iterative fashion by using the alternating combinations of the SpyCatcher and SnoopCatcher systems flanking the antigen proteins. Combinations of antigens to form multivalent OMV-based vaccines can significantly improve their efficiency, either by eliciting a stronger immune response to a particular pathogen, or by providing a broader response by combining antigens from multiple pathogens [94,95]. For this approach, they treated the OMVs with PspAα fusion and a SnoopCatcher–SP1690–SpyTag fusion with wash steps in between, successfully forming bipartite (Figure 7B) and tripartite (Figure 7C) coupling adducts. This work demonstrated how various recombinant antigens can be coupled to the OMV surface using the SpyCatcher system.

A similar application for the SpyCatcher system in vaccine development has been used for VLPs. VLPs are virus-derived proteins that self-organize into noninfectious virus-like structures that are capable of eliciting strong immune reactions [96]. Much effort has been put into designing VLP–antigen fusions; however, this approach has proven to be labor-intensive and time-consuming, since the fusions often misalign, or are unable to form stable capsids [97]. Brune et al. from the Howarth group circumvented this problem by using the SpyCatcher technology in an analogous fashion to the OMV-based vaccines [44]. They were able to create stable SpyCatcher-presenting VLPs by using a fusion between the bacteriophage AP205-derived capsid and SpyCatcher. They further demonstrated how the VLPs could be decorated using SpyTag-fused malarial antigens (Figure 2F) in a plug-and-play fashion, and how this product elicited an antibody response after a single injection in mice. In similar work, Thrane et al. fused either SpyTag or SpyCatcher to the C- and/or N-termini of the AP205 capsid protein, and subsequently treated the resulting VLPs with 11 different antigen–SpyCatcher/SpyTag fusions [43]. In both works, the authors confirmed the immunogenic properties of the novel vaccines by immunizing mice, thus confirming the functional activity of the novel vaccines. The results from the OMV and VLP experiments demonstrated the versatility of the SpyCatcher system in a novel approach for designing vaccines. By presenting one of the binding SpyCatcher/SpyTag partners on the particle surface, linking antigens in a plug-and-play fashion circumvents the need for designing complicated fusion constructs, and could soon pave the road for modular vaccines that can easily be tailored for any need. For further information, Brune and Howarth recently published a more comprehensive review on the design of VLP-based vaccines, including the use of the SpyCatcher platform for this purpose [14].

## 4. Conclusions and Future Perspectives

The SpyCatcher system and its close relatives have been successfully used for protein engineering purposes, including the cyclic polymerization of enzymes, or the decoration of hydrogels. In our hands, the system has been extremely useful for showing cell surface localization, and to elucidate the topology of membrane proteins. Other authors have used the technology for decorating vesicles and virus particles with antigens. In all cases, the benefits of the system are the same: irreversible, covalent coupling and the fact that the two partners of the system are relatively small entities that do not interfere strongly with the native systems that they label.

It is somewhat ironic that the system, derived from a bacterial surface protein, has been widely used for studying and modifying other bacterial or viral surface proteins. We strongly believe that this system will have many more applications in basic science, and that this is only limited by the fact that many researchers are not aware of its existence. This prompted us to write this review.

Some of the approaches related to vaccines will need more testing before they can potentially be used in patients. For example, it remains unclear whether SpyCatcher (or its relatives) elicits a strong immune response by itself, and whether that would limit its usefulness in vaccines. The work on VLPs suggests that this could be problematic for some applications, where the SpyCatcher-VLPs did elicit an immune response of their own that was then later masked after decorating the particles with the target antigen [44].

Our own experiments were until now limited to surface-localized proteins in bacteria, but it is conceivable that the system could also be used to label proteins in other cellular localization (e.g., after membrane permeabilization) as an alternative to antibody-based approaches. We also believe that the system can be used in versatile ways to modify and functionalize artificial or biological surfaces of almost any kind (e.g., to develop ELISA-like assays that require covalent immobilization), and possibly to functionalize surfaces used in lab-on-a-chip approaches. Another conceivable use of the system includes in situ labeling of proteins for added density to identify individual proteins (e.g., in cryo-electron micrographs or tomograms). As this review shows, the SpyCatcher-SpyTag system has proven to be an extremely versatile tool for both basic research and applied science, and it will undoubtedly spur further innovations in the future.

## Figures and Tables

**Table 1:** Summary of Catcher-Tag technologies and their development.

Catcher	Tag	Tag Sequence	Description	Publication Year	Reference
SpyCatcher	SpyTag	AHIVMV**D**AYKPTK ^1^	Original Catcher-Tag technology.	2012	[9]
SpyCatcher ΔN1ΔC1	SpyTag	AHIVMV**D**AYKPTK ^1^	Minimal SpyCatcher construct that still binds efficiently to SpyTag.	2014	[20]
SpyLigase ^2^	SpyTag	AHIVMV**D**AYKPTK ^1^	Rationally engineered system for ligating two peptides.	2014	[18]
	KTag	ATHIKFS**K**RD			
SnoopCatcher	SnoopTag	KLGDIEFI**K**VNK ^1^	Orthogonal technology to SpyCatcher.	2016	[16]
SpyCatcher002	SpyTag002	VPTIVMV**D**AYKRYK ^1^	Improved SpyCatcher-SpyTag system with faster reaction rate.	2017	[21]
SnoopLigase ^2^	SnoopTagJr	KLGSIEFI**K**VNK ^1^	Rationally engineered system for ligating two peptides.	2018	[19]
	DogTag	DIPATYEFTDGKHYIT**N**EPIPPK			
SpyDock	SpyTag002	VPTIVMV**D**AYKRYK ^1^	Protein affinity purification system (Spy&Go) based on SpyCatcher.	2019	[22]

^1^ The reactive residues are shown in bold. ^2^ SpyLigase and SnoopLigase have two target peptides.

## Abbreviations

CLIP	UV crosslinking and immunoprecipitation
GoldCLIP	Gel-omitted ligation-dependent CLIP
Hbp	Hemoglobin protease
MSCRAMMs	Microbial surface components recognizing adhesive matrix molecules
OM	Outer membrane
OMV	Outer membrane vesicle
PDB	Protein Data Bank
SDS-PAGE	Sodium dodecylsulfate polyacrylamide gel electrophoresis
sfGFP	Superfolder green fluorescent protein
SUMO	Small ubiquitin-like modifier
TAA	Trimeric autotransporter adhesin
UV	Ultraviolet
uvCLAP	UV-crosslinking and affinity purification
VLP	Virus-like particle
YadA	*Yersinia* adhesin A
YadAM	YadA membrane anchor
